# Iron Supplementation during Pregnancy and Infancy: Uncertainties and Implications for Research and Policy

**DOI:** 10.3390/nu9121327

**Published:** 2017-12-06

**Authors:** Patsy M. Brannon, Christine L. Taylor

**Affiliations:** 1 Division of Nutritional Sciences, Cornell University, Ithaca, NY 14853, USA; 2 Office of Dietary Supplements, National Institutes of Health, 6100 Executive Blvd, 3B01, Bethesda, MD 20892, USA; TaylorCL3@od.nih.gov

**Keywords:** iron supplementation, iron-replete, pregnancy, infancy

## Abstract

Iron is particularly important in pregnancy and infancy to meet the high demands for hematopoiesis, growth and development. Much attention has been given to conditions of iron deficiency (ID) and iron deficient anemia (IDA) because of the high global prevalence estimated in these vulnerable life stages. Emerging and preliminary evidence demonstrates, however, a U-shaped risk at both low and high iron status for birth and infant adverse health outcomes including growth, preterm birth, gestational diabetes, gastrointestinal health, and neurodegenerative diseases during aging. Such evidence raises questions about the effects of high iron intakes through supplementation or food fortification during pregnancy and infancy in iron-replete individuals. This review examines the emerging as well as the current understanding of iron needs and homeostasis during pregnancy and infancy, uncertainties in ascertaining iron status in these populations, and issues surrounding U-shaped risk curves in iron-replete pregnant women and infants. Implications for research and policy are discussed relative to screening and supplementation in these vulnerable populations, especially in developed countries in which the majority of these populations are likely iron-replete.

## 1. Introduction

Iron has long been recognized as essential, but its nutritional status is nonetheless characterized by many challenges and unknowns, especially when the focus is pregnancy and infancy. Iron plays key roles in oxygen transport by red blood cells (RBC), energy production, growth and development, functions particularly important during the demands in pregnancy and infancy for hematopoiesis, growth and development. Much attention has been given to conditions of iron deficiency (ID) during these vulnerable life stages, but more recently questions have arisen about the effects of iron supplementation when individuals are iron-replete. Resolving these questions in general as well as in the case of pregnancy and infancy requires better understanding of iron homeostasis, biological adaptations, approaches to determining iron status, and the risk from not only too little but also too much iron.

The highly reactive chemical nature of the iron molecule, particularly its redox chemistry [1] and interaction with oxygen [2], underlie both its essential functions and cytotoxic actions. Its ability to form iron polymers through hydroxide complexes is also important for its storage complexed to the protein, ferritin. In physiologic concentrations, iron functions in both oxygen transport and energy production through its redox potential. In excess, however, iron is a pro-oxidant and produces reactive hydroxyl radicals and other reactive oxygen species (ROS) that damage DNA, proteins, lipids, other cellular molecules and stem cells [2]. Thus, ensuring adequate availability, but not excess, drives iron homeostasis. Iron exhibits a U-shaped nutrient-health relationship because of functional impairment when inadequate and cytotoxicity when excessive. This duality of its effect on health reflects a continuum of iron status from frankly deficient to inadequate stores to replete stores to high stores to toxic levels.

ID, well recognized as a public health concern, results when iron stores are inadequate to meet tissue needs and culminates in iron deficiency anemia (IDA) and fatigue when stores are fully depleted and erythropoiesis is impaired. The risk of ID or IDA increases in physiologic states of high blood loss (reproductive aged women) or increased physiologic need (pregnancy and infancy). ID and IDA receive greater attention than iron excess, both in research and policy, because of their high global prevalence, especially in developing countries.

Globally, over 40% of pregnant women and 47% of preschool children are anemic from all causes [3]. The World Health Organization (WHO) estimates that 50% of these anemias are due to ID and reflect IDA. However, Petry et al. [4] recently suggested based on their systematic review and meta-analysis of nationally representative survey data for preschool children and non-pregnant women that only about 25% of such anemia overall is attributable to ID. Although the global prevalence of IDA has not been measured directly, 10 to 20% of pregnant women and 15 to 24% of school-aged children are likely to have IDA when WHO estimates are reconsidered in light of the recent evidence from Petry et al. The WHO recommends universal iron supplementation [5,6] for pregnant women and young children 6 to 24 months because of this high prevalence of IDA.

However, the environmental and health context for developed countries differs because the prevalence of ID and IDA is lower. The most recent analysis of 1999–2010 National Health and Nutrition Examination Survey (NHANES) data found a prevalence of 2.6% and 2.2% IDA in pregnant women and young children (12–23 months) in the United States [7]. The prevalence of ID was 16.3%and 15.1% in pregant women and young ehildren with a significantly higer prevalence among Non-hispanic black, Mexican American and low-income pregnant women. The prevalence of IDA varies from a low of 3% in Switzerland to 15% in Belgium in European pregnant women [8] and below 5% in Northern and Western Europe in European young children [9]. Despite the lack of established cutpoints for iron-replete status, it appears that the majority of these populations in developed countries are likely to be iron-replete [10] even among ethnic and low socioeconomic individuals in whom the prevalence of ID may be higher than the general population.

Concerns exist and continue to emerge, as discussed in detail below, about the risk of adverse outcomes including growth, gestational length, gestational diabetes mellitus, and gastrointestinal health [11,12,13,14,15] with high intakes and iron status during pregnancy and infancy. Physiologic or developmental adaptation of iron homeostasis appears to occur in pregnancy [11] and infancy [12] to meet the higher physiologic needs for iron during these periods, but these adaptations, along with the lower loss of iron due to the cessation of menses during pregnancy, may potentially enhance the vulnerability to high iron intakes in iron-replete individuals.

This review examines the emerging as well as the current understanding of iron needs and homeostasis during pregnancy and infancy, uncertainties in ascertaining iron status in these populations, and issues surrounding U-shaped risk curves in iron-replete pregnant women and infants. It concludes with a discussion of the implications for research and policy relative to screening and supplementation in these vulnerable populations.

## 2. Iron Needs: Considerations during Pregnancy and Infancy

The physiologic demand for iron is especially high in pregnancy and infancy with an estimated 1000–1200 mg of iron needed during pregnancy [8,11]. About two thirds of this iron is for maternal needs, and 1/3 is for placental-fetal tissue needs [11]. However, the need varies across gestation with lower need in the first trimester (0.8 mg/day) than the need before pregnancy and much higher need in the third trimester (3.0–7.5 mg/day) [13]. This progressive increase reflects the temporal pattern of hematopoiesis and fetal growth [11]. Maternal hematopoiesis and RBC expansion as well as fetal growth are much higher in the second half of pregnancy. Thus much of the 330–400 mg for fetal growth is, therefore, needed in the last trimester. Some of the total iron need may be met by maternal iron stores in iron-replete women, and approximately 300 mg of this total iron is recycled and again available to the mother as her RBC volume contracts postpartum [16]. About 750 mg of additional iron is needed during pregnancy beyond that mobilized from and then returned to maternal stores in iron-replete women. For women with low or depleted iron stores, 1000 mg or more of additional iron might be required to meet maternal and fetal iron needs during pregnancy.

Despite this progressive physiologic need, established reference intake values for iron in several developed countries average the need for iron across pregnancy rather than varying requirements by trimester. The United States, Canada, Australia and New Zealand recommend 150% higher intakes for pregnant women than for reproductive age women beginning at the initial stages of pregnancy (Table 1). However, the United Kingdom and Europe do not identify a need for an increase during pregnancy, and WHO does not specify intakes for pregnancy. Consequently, the reference intake values vary internationally. Specifically, the Estimated Average Requirement (EAR) or Average Requirement (AR) varies from 7 mg/day to 22 mg/day to meet the needs of 50% of the population, and Recommended Daily Allowance (RDA) or Population Nutrient Intake (PNI) or Recommended Nutrient Intake (RNI) varies from 11.5 mg/day to 27 mg/day, to meet the needs of 97.5% of the population (see Table 1).

After birth, the need for iron is met primarily from iron stores in the exclusively breastfed full-term infant for the first 4 to 6 months because human milk is low in iron even though this iron is highly bioavailable. These iron stores derive from a portion of the approximate 270–330 mg of iron transferred in utero. Typically, such infants have approximately 80 mg of iron per kg [12,22] and an initially higher hemoglobin (Hb) concentration of about 17.0–19.0 g/dL [23]. Hb concentration steadily declines in the first 12 weeks [23], and iron is scavenged from the degraded Hb. The magnitude of iron stores, however, depends on the iron status of the mother, the gestational age at birth, the delay in cord clamping, and the birth weight of the infant. Thus, preterm or small for gestational age (SGA) infants and infants born to women with IDA are more likely to have lower iron stores that may be depleted earlier than 4 to 6 months [12]. Established reference intake values are low in the first six months when iron needs are met by stores (Table 1). Several developed countries recommend an Adequate Intake of 0.2 to 0.26 mg/day (United States, Canada, Australia and New Zealand), but the United Kingdom recommends a higher RNI (1.7 to 3.3 mg/day for 0–3 and 4–6 months). After iron stores are depleted, dietary iron needs increase to meet the sustained high demands for hematopoiesis, tissue accretion and brain development. Some suggest that during human evolutionary history this additional dietary iron may have come from pre-mastication of meat, a source of highly bioavailable heme iron [24]. Currently, the American Academy of Pediatrics encourages early introduction of pureed meat as a highly bioavailable source of heme iron [25], but intakes of meat by infants are low in the United States [26]. Canadian and Australian health authorities also recommend iron-rich complementary foods including meat (Table 2). Most developed countries (US, Canada, UK, Australia, New Zealand and Europe) recommend an EAR/AR of 6–8 mg/day and an RDA/PNI/RNI of 7.9–11 mg/day for older infants (6 to 12 months) and an EAR/AR of .3–5.3 mg/day and an RDA/PNI/RNI of 6.9–9 mg/day for young children (12 to 23 months). 

Recommendations for supplementation vary across developed countries (Table 2) with no routine supplementation recommended for pregnant women and young infants by Canadian and Australian health authorities and for young infants by the European Society Pediatric Gastroenterology, Hepatology and Nutrition but universal supplementation for pregnant women and breast-fed infants four months and older by the United States Center for Disease Control and the American Academy of Pediatrics, respectively. Other authoritative groups in Europe, Britain and New Zealand (Table 2) recommend screening of pregnant women and supplementation if “at risk” or evidence of ID is found. The United States Preventative Services Task Force’s recent finding of insufficient evidence for or against universal iron screening and supplementation of pregnant women [27] and screening of young children 6 to 24 months [28] underscores the need for research to inform policy and practice decisions to ensure adequate iron status in these vulnerable populations in developed countries.

## 3. Iron Homeostasis: Physiologic and Developmental Adaptations during Pregnancy and Infancy

Iron homeostasis is the coordinated process through which key proteins regulate iron absorption, recycling, transport and storage to ensure iron availability without excess. The hepatic protein, hepcidin, functions as a master regulator in this homeostasis through its down-regulation of intestinal and tissue release of iron. It interacts with the cellular iron exporter, ferroportin (FPN) to reduce iron efflux and, thus, availability. When iron stores and availability are low, hepcidin is low; and more iron is released from that absorbed in the intestine or stored in tissues. In contrast when iron stores and availability are high, hepcidin is elevated; and less iron is released from that absorbed in the intestine or stored in tissues [38]. Hepcidin is also upregulated by inflammation and infection to sequester iron stores and reduce iron absorption as a part of anti-infective responses [39] and downregulated by hypoxia and erythropoiesis to meet iron needs. Mutations in selected key regulatory proteins that interact with hepcidin impair this homeostasis and result in hemochromatosis characterized by iron overload and cytotoxicity [40]. Allelic variants in selected iron regulatory or transport proteins also appear to enhance susceptibility to either high iron stores or ID in some ethnic sub-populations [40]. 

Adaptations in this iron homeostasis have been suggested both in pregnancy and early infancy, most likely to meet the substantive needs for iron during these periods. In pregnancy, physiologic adaptations appear to increase iron absorption [11]. Although the mechanism of this adaptation is not well understood, iron homeostasis appears to be “reset” through suppression of hepcidin, even though hepcidin still responds to iron availability, erythropoiesis, inflammation and hypoxia albeit at a “blunted” level [11]. Irrespective of maternal or fetal iron status [41], hepcidin concentrations decrease during pregnancy to nearly undetectable levels in the latter half of pregnancy Nemeth has proposed that an as-yet unidentified regulatory factor reduces the regulatory responsiveness of hepcidin to a lower level [11]. 

Developmental adaptations may also alter the homeostatic regulation of iron absorption by hepcidin in the young infant. Although both hepcidin and its target FPN are present [12], limited preliminary evidence suggests that iron absorption may not be regulated by iron status or supplementation in the infant prior to 6 months [42] or in the suckling-only rat pup prior to day 10 [43,44]. Later in the older human infant and rat pup with complementary feeding or nibbling-suckling transitional feeding, iron absorption does exhibit its usual homeostatic regulation in response to hepcidin. The mechanisms of this attenuated iron homeostatic regulation are largely unknown [12]. One potential consequence of this apparent developmental adaptation is high absorption of the limited amount of iron in human and mammalian milk during the suckling-only period. Conceivably, evolution of infants and other mammals led to unrestrained absorption of limited dietary iron [10]. 

The relevance of these physiologic and developmental adaptations of iron homeostasis is not understood, but these may serve to facilitate iron availability during peak periods of erythropoiesis. Given the widespread recommendation and use of iron supplements in both pregnant women and infants and iron-fortified formula in developed countries, however, these adaptations may also limit the primary protective mechanisms against excessive iron uptake. What is even less well understood is the extent to which these might, therefore, enhance the risk of high or excess iron status in the context of largely iron-replete populations [38]. Also unknown is how effectively these adapted homeostatic systems respond to key regulators such as iron status and inflammation although in pregnancy some limited evidence suggest that they do. Equally unclear is the extent to which maternal baseline iron status influences the resetting of maternal iron homeostasis [11].

In addition to the physiologic and developmental adaptations of this homeostatic regulation, developing tissues in the fetus and infant differentially acquire iron [45], such that hematopoietic needs are met before the needs of critical tissues such as the brain. In the context of sufficient iron, this does not limit availability to all developing tissues. However, in the context of limited iron availability, the brain can experience ID and permanent damage without impairment of hematopoiesis and hematologic indicators of iron status [45]. The mechanisms of this differential prioritization are also not understood, but clearly have implications for assessing iron status with the common hematologic indicators in the young child through 24 months, the period of rapid and critical brain development and high brain iron needs.

In summary, adaptations in iron homeostasis appear to occur physiologically in pregnancy and developmentally in young infants. They enhance iron absorption, but may limit feedback regulation in response to high iron status. The mechanisms of these adaptations are unknown. Moreover, the extent to which they might also enhance or not susceptibility to excessive absorption with high supplementation or iron-fortification is not understood.

## 4. Iron Status: Uncertainties in Assessment of Pregnant Women and Infants

### 4.1. Commonly Used Indicators

Assessing iron status is complex at any life stage, in part because no single indicator is sufficiently specific or sensitive to be used alone; thus, multiple indicators must be measured and integrated to estimate iron status. The most commonly used indicators are presented in Table 3. All are primarily hematologic indicators, but differ where they assess the continuum of iron status from iron stores to tissue depletion to impaired erythropoiesis to anemia resulting from impaired erythropoiesis. 

Uncertainties exist in assessing iron status in pregnant women and infants, as well as in other life stages, because of analytic issues including lack of standardization of the assays; confounding especially by inflammation; and lack of established health outcomes relative to cutpoints. Unique to pregnancy are physiologic changes due to plasma volume expansion resulting in hemodilution and a mild inflammatory state [16]. The extent to which this affects common indicators has only been partially assessed for Hb concentration. Collectively, these uncertainties may result in misclassification of ID and IDA, introducing further uncertainties in estimating the prevalence of ID and IDA. In addition, the full continuum of iron status including iron repletion and excess cannot be determined because there are no cutpoints for common iron indicators for repletion and excess. This is true even for Total Body Iron stores (TBI, the log ratio of serum ferritin (SF) to soluble transferrin receptor (sTfR), which theoretically can be used to assess the full continuum of iron status, but in practice is limited by the lack of established cutpoints for repletion and excess. Finally, iron status, especially the level of stores which are essential to assess, change throughout the course of pregnancy as stored iron is mobilized to meet the high demand. Few studies have examined longitudinally the physiologic use of stores in iron-replete women, and none have assessed longitudinally repletion of iron stores postpartum.

### 4.2. Analytic Challenges

The analytic issues surround the lack of harmonization and standardization of the indicator assays [46]. The available WHO international standard material for SF concentration derives from “consensus” values because no standard reference method exists. This will also be the case for the other WHO international standard material for sTfR in development. The prospect for developing standard reference methods for SF and sTfR is poor because of the size of the proteins involved and technical challenges inherent in doing so [46,47]. In the absence of such standardization, measures may exhibit imprecision and inflated Confidence Intervals [46,48] that can create interpretative challenges and also contribute to misclassification of ID and IDA. 

### 4.3. Confounding

Exacerbating these uncertainties is the documented confounding of SF and to a lesser extent sTfR, as well as indicators based on these two measures such as TBI, by inflammation. The Biomarkers Reflecting Inflammation and Nutrition Determinants of Anemia (BRINDA) project reports linear regression algorithms to adjust SF based on cross-sectional data from multinational population indicators of acute (C-reactive protein) and chronic (alpha1-acid glycoprotein) inflammation [49,50,51]. Application of these proposed adjustments to TBI in U.S. women of reproductive age increased slightly by 7 percentage points the prevalence of ID [7]. However, none of these algorithms derive from analysis of data from pregnant women, who experience a mild inflammatory state due to pregnancy itself In addition, hepcidin concentration and inflammation during pregnancy do not appear to correlate [11], but some caution is warranted as this relationship has yet to be evaluated in infection or severe inflammation in pregnant women. Emphasizing the uncertain feasibility of adjusting for inflammation in pregnancy is the lack of correlation of inflammatory and iron status indicators in pregnant adolescents except at delivery [52]. Further, the BRINDA algorithms do not derive from analysis of data for young children (0–24 months). The relationship remains unknown between iron status and inflammatory indicators during pregnancy and young children, but the nature of these relationship needs to be evaluated in order to assess whether there may be ways to adjust for inflammation during pregnancy.

### 4.4. Linking Measures to Health Outcomes

A major limitation in, and the resulting uncertainty for, current cutpoints stem from the lack of their established relationships to non-hematologic or clinically relevant health outcomes in pregnant women, infants and young children for all common measures of iron status. Currently, cutpoints for pregnancy and young children derive from the lowest percentile distribution of the population, typically below the 5th percentile for Hb concentrations for anemia and, thus, IDA. For pregnant women, the Centers for Disease Control and Prevention (CDC) trimester-specific cutpoints were informed by four small longitudinal studies from 30 years ago [52], These trimester-specific cutpoints do consider hemodilution due to plasma volume expansion, but today’s gynecologic population is older and has greater adiposity and incidence of gestational diabetes as well as higher mortality [16]. All of these factors in today’s population may affect plasma volume expansion and hemodilution, such that the distribution of common indicators longitudinally in current populations may differ from those upon which these cutpoints were based. Thus, longitudinal studies in today’s gynecologic population are needed. Further, cutpoints for SF are the same in pregnant women as those for reproductive age women, ranging from <10 to <15 µg/L and varying among clinical laboratories [8,46,53]. Therefore, the cutpoints for SF do not consider hemodilution due to plasma volume expansion or longitudinal physiologic changes in SF documented in iron-replete pregnant women. Whether TBI, is more independent of plasma volume expansion needs to be evaluated because TBI was only evaluated in a small number of adult males and non-pregnant females [47]. In addition to these limitations in the current population-based cutpoints and lack of evaluation in pregnancy or infancy for SF and TBI, the United States Preventive Services Task Force (USPSTF) noted an additional uncertainty because evidence is lacking on whether changes in these hematologic indicators in pregnant women, infants or young children reflect “meaningful improvements in health outcomes” beyond the hematologic outcome of anemia [54].

Evaluation of these indicators relative to health outcomes, especially non-hematologic outcomes, could inform cutpoints with clinical and public health relevance for the full spectrum of iron status [53]. In particular, TBI may be a useful indicator as it has the potential to be related to the full continuum of iron status. Such evaluation might, in fact, need to use different outcomes for assessing high and low exposure. The relationship of common iron indicators to meaningful outcomes across the full continuum of iron status is also important for developing stronger evidence-based clinical and public health guidelines to ensure adequate iron status and, thus, ensure normal development of critical tissues. Such evaluation based on non-hematologic outcomes is also important for monitoring supplementation, especially during pregnancy, infancy and young childhood when iron is differentially prioritized to erythropoiesis. Indicators that only assess hematologic outcomes fail to identify partially depleted iron stores that could adversely affect critically developing tissues such as the heart brain when hematopoiesis is not yet affected. To improve screening and monitoring, we need indicators that are particularly informative of tissue ID in pregnancy, infancy and young children.

Collectively, the lack of standardization and harmonization of assays, confounding by inflammation, impacts of physiologic changes on the indicators and lack of established relationship with non-hematologic outcomes introduce uncertainty in the measurement and interpretation of iron indicators in pregnant women, infants and young children. These uncertainties increase the risk of misclassification of ID and IDA and also limit interpretation of screening and monitoring of these vulnerable populations. 

## 5. U-Shaped Risk for Iron Status: Concerns for Pregnant Women and Young Infants

Iron exhibits a U-shaped risk, typical of essential nutrients, in which risk of adverse outcomes in pregnant women, infants and young children increases not only with low or inadequate availability but also at higher availability [55,56]. Research and public health programs have focused primarily on the increased risk with low availability, particularly in pregnant women, infants and young children because of the high global prevalence of low iron status, namely ID and IDA. However, emerging evidence calls attention to possible increased risk of adverse outcomes with higher iron status. The 2016 USPSTF finding of insufficient evidence for or against iron screening and supplementation in these populations prompted the National Institutes of Health (NIH) Office of Dietary Supplements to hold a workshop on Iron Screening and Supplementation in Iron-Replete Pregnant Women and Young Children in September 2016 to consider the evidence of risk in iron-replete populations and identify research needs. During the workshop, the clear concern for increased risk of adverse outcome with ID and IDA was evident, but so, too, were the uncertainty and concern for the potential risk for adverse outcomes with high iron status. The physiologic need for iron and apparent adaptations of iron homeostasis as well as common and often routine supplementation of largely iron-replete pregnant and young children in developed countries underlay these concerns as well. The nature of the evidence for this U-shaped risk with iron and the uncertainties of this evidence are worthy of consideration. 

### 5.1. Left-Side of U-Shapes Risk Curve: Low Iron Status

In terms of low iron status during pregnancy, most of the evidence has examined the relationship of Hb concentration or anemia and increased risk of maternal and fetal adverse outcomes without consideration of concomitant inflammation [56]. Low Hb concentrations associate with increased risk of low birthweight (LBW), small for gestational age (SGA), and preterm births [55,56] and maternal mortality [55], but closer examination of the synthesized evidence reveals that this association holds for LBW and preterm birth with low Hb concentrations in the first trimester, but not the second or third trimesters [56]. Although variable cutpoints for anemia and low Hb concentrations were used across the studies, a meta-analysis in 2012 found that the increased risk of SGA associated only with moderate to severe anemia [57] or Hb concentrations <9 g/dL for SGA and preterm births [55]. However, many factors can cause anemia, but only a few studies have examined the relationship of IDA, low SF and high sTfR with adverse pregnancy outcomes and reported less consistent findings than those examining the relationship of low Hb concentrations. Only one of two studies in the first trimester and one of three studies in the second trimester found an association of low SF with SGA or preterm birth and LBW, respectively [56]. Dewey also reported that the higher iron status (lower sTfR or higher SF within normal ranges) early in pregnancy generally associated with better birth outcomes in three cohorts in Ghana, Malawi and Bangladesh, whereas later in pregnancy it did not. Despite the limited evidence and lack of control for confounding by inflammation, the current evidence does suggest s increased risk of maternal and fetal adverse outcomes with anemia and low iron status as assessed by SF and sTfR, particularly early in pregnancy. 

ID even more so than IDA is a concern in the infant and young child because of the vulnerable period of brain development in the first 24 months. As discussed earlier, the differential prioritization of iron to erythropoiesis exacerbates the vulnerability of the brain when iron is limiting during this critical period. A limited number of studies find impaired cognitive and brain development with ID [45,58,59,60]. Nonetheless, the importance of adequate iron is emphasized by this evidence relative to this vulnerable period in the infant’s and young child’s brain development and likelihood of lasting impairment without adequate iron. Further emphasizing the importance of sufficient iron during this period is a recent systematic review that reports improved psychomotor development in young infants < 6 months exclusively breastfed and supplemented with iron [61] without improvement of ID, IDA, or SF. 

Another important evaluation of the risk of low iron status is improvement in outcomes with iron supplementation of pregnant women, infants and young children with low iron status. Although such supplementation generally improves hematologic indicators, which can be viewed as intermediate outcomes for anemia [53,54,62], the benefits and harms of routine iron supplementation on other non-hematologic health outcomes is uncertain. Both the USPSTF and the updated 2011 Cochrane review [63] noted the lack of quality studies reporting on clinical or health outcomes. A meta-analysis in both developed and developing countries, however, reported improved birthweight in a linear dose-response relationship and maternal Hb concentrations in the third trimester with daily iron supplement use in both high, middle and low income countries [64]. In contrast, a recent systematic review of iron supplementation of young children (6–24 months) in developed countries found no clear benefit for growth outcomes (5/6 trials) or infant development in the first 12 months (2 trials), inconsistent findings for improvement of hematologic outcomes and no reports in any of the identified studies related to neurodevelopmental delay or improvement of hematologic indicators and clinical outcomes [65]. As noted previously, the 2017 systematic review [61] found only four randomized clinical trials (RCTs) on iron supplementation of young exclusively breastfed infants <6 months and reported both benefit in terms of psychomotor development and harm in terms of reduced growth without consistent improvement of iron status in terms of IDA, ID, or SF. In spite of the need for more evidence from well-controlled RCTs especially evaluating non-hematologic health outcomes and the relationship of improved iron status with supplementation and health outcomes, all agree that treatment of ID and IDA during pregnancy and young children is important and warranted.

### 5.2. Right-Side of U-Shaped Risk Curve: High Iron Status

High iron status during pregnancy also associates with increased risk for maternal and fetal adverse outcomes. Across studies, high Hb concentrations, especially in the second trimester, associate with LBW as comprehensively reviewed by Dewey and Oaks [56] and Breymann [55], but has an inconsistent in relationship with preterm birth or SGA [56]. High SF concentrations also associates with increased risk for LBW or preterm birth [56] and SGA [55]. Dewey and Oaks also reported that higher iron status associated with lower birth size in some of the cohorts in Ghana, Malawi and Bangladesh [56]. Again, most of the studies have focused on the relationship of high Hb concentrations and high SF and adverse birth outcomes without consideration of inflammation or impaired plasma volume expansion, which is a major and serious limitation of the evidence to date. Scholl emphasizes the concern that high Hb may reflect impaired plasma volume expansion or inflammation due to infections [66]. Future studies need to consider carefully plasma volume expansion and the presence of inflammation in evaluating the relationship of high iron status assessed by Hb or SF concentrations. 

In addition, preliminary evidence also links supplementation or high iron status to emerging adverse outcomes in pregnancy including gestational diabetes mellitus (GDM) in observational case-control and prospective cohort studies and limited RCTs [14]. At present, the evidence for GDM is inconsistent and limited by predominant assessment of high iron status by high Hb or SF concentrations without consideration of concomitant inflammation. Further, GDM itself is associated with inflammation, making it essential to assess acute and chronic inflammation in evaluating the linkage of high iron status with GDM. Although the mechanism is unknown, one possibility may be ROS and damage of pancreatic β cells resulting in diabetes [14]. Further research is needed to clarify the relationship of high iron status and supplementation of iron-replete pregnant women to the risk for GDM. 

Iron supplementation of iron-replete infants and young children increased the risk of vomiting and fever in a systematic review of the evidence [67] and altered microbiome profiles in Tanzanian children [15] and impaired linear growth in Swedish infants [68]. In contrast, consumption of iron-fortified formula (9–12 mg/L) by older infants in the United Kingdome from 9 to 18 months did not affect infections, gastrointestinal problems or weight gain, but linear growth was not measured [69]. Although the mechanism whereby iron supplementation of iron-replete young children could adversely impact the gastrointestine and its microbiome are not known, most of the iron in a supplement is not absorbed and could promote a more pathogenic microbiome that depend on iron with resulting diarrhea. However, the environmental context may influence such a response depending on the overall risk for infection. In terms of impaired linear growth, Lonnerdal has proposed that iron supplementation of iron-replete infants might impair such growth through interactions of excess iron with zinc or copper [68]. Nonetheless, further research evaluating the potential adverse effects of iron supplementation of iron-replete young children particularly in developed countries is needed to clarify the risk and its nature to evaluate the risk relative to benefit. Given the observational nature of much of this emerging evidence, strongly designed studies are needed to determine the causality of these relationships. 

Another proposed adverse outcome of high neonatal exposure relates to cumulative high brain iron and neurodegeneration in older adults [70]. Supportive evidence in humans includes the association of high iron concentrations in the brain with neurodegenerative diseases such as Parkinson’s and Alzheimer’s from primarily from case-control studies [2,70]. A systematic review of cross-sectional epidemiologic studies reports inconsistent association of measures of iron status with cognitive impairment [70]. However, preclinical studies of neonatal iron supplementation in rodent models identified in this systematic review report increased brain iron later in life with adverse effects on brain morphology and biochemistry in a variety of areas. Possible mechanisms include ROS damage to brain cells and to stem cells [2], but remain unknown. These preliminary findings and proposed relationship of early high iron exposure and subsequent neurodegenerative disease raise the possibility of Developmental Origins of Adult Disease (DOHaD) in relation to excess early iron. 

Overall, current evidence supports a U-shaped risk curve for a variety of adverse birth outcomes in mother and neonate as well as in young children. Emerging evidence also raise concerns about adverse short-term and long-term health outcomes with iron supplementation of iron-replete pregnant women and children. Uncertainties in this evidence, particularly the failure to consider concomitant inflammation and limited evidence for non-hematologic health outcomes, emphasizes the need for well-controlled cohort longitudinal studies and RCT to evaluate the benefits and harms of iron supplementation in iron-replete populations. 

## 6. Implications for Research and Policy

**Figure 1:**
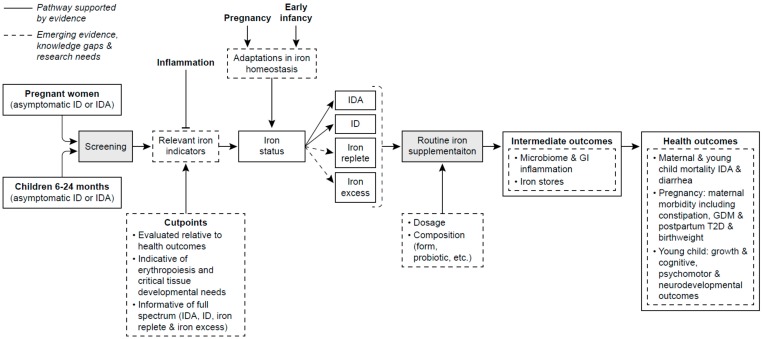
Analytic framework for iron screening and supplementation of pregnant women and young children in developed countries. Solid lines highlight pathways supported by current evidence. Dashed lines highlight emerging evidence, uncertainties and research needs. Abbreviations include ID, iron deficiency; IDA, iron deficient anemia; GI, gastroinestine; GDM, gestational diabetes; T2D, type 2 diabetes. (From Brannon et al. [48] and reprinted with permission by American Journal of Clinical Nutrition: Am. J. Clin. Nutr. 2017; 106(Suppl): 1703S–12S. Printed in USA. © 2017 American Society for Nutrition).

A number of knowledge gaps and research needs were identified at the NIH Workshop on Iron Screening and Supplementation of Iron-Replete Pregnant Women and Young Children in 2016. Four major themes emerged from these gaps and research needs focusing on (1) elucidating adaptations of iron homeostasis in pregnancy and early infancy including their mechanisms, responsiveness to iron status, interaction with genetic and ethnic factors and implication’s for the differential prioritization of iron to developing tissues and hematopoiesis; (2) improving the assessment of iron status particularly in these two vulnerable periods including the measurement uncertainties, need for indicators across the full continuum of iron status that are adjustable or independent of inflammation; (3) evaluating iron status relative to maternal and infant health outcomes, especially non-hematologic outcomes; and (4) determination of short and long term beneficial and adverse health outcomes, especially non-health outcomes such as GDM, LBW, SGA, postnatal growth and DOHaD, with iron supplementation of iron-replete pregnant women and young children [45,48]. In Figure 1, an analytic framework of these knowledge gaps and research needs is depicted along the pathway from screening to supplementation to outcomes. This pathway is based on current evidence (shown in solid lines) with the research needs and gaps of knowledge (shown in dashed lines). 

Illustrated from this framework and the discussion above of the limitations of the evidence even from the benefit of improved hematologic outcomes with supplementation is the limited and preliminary nature of the current evidence. Clearly, well-controlled longitudinal cohort studies in iron-replete and generally well-nourished populations in developed countries are needed to understand the dynamic mobilization of iron stores and its impact on clinically-relevant and non-hematologic health outcomes. So too, are well-designed RCTs examining both the benefits and potential harms of iron supplementation on such health outcomes.

Importantly, little if any research has considered the interaction of baseline iron status (and level of stores) and the beneficial or harmful response to iron supplementation. Critical to understand however regarding this interaction is the determination of who is at risk of adverse outcomes from low iron status and would benefit from iron supplementation. A parallel consideration is the amount and form of iron that is most effective with least adverse effect for either supplementation or food fortification. Based on emerging data, the interaction of the level of iron stores among iron-replete pregnant women and infants with high dietary iron intakes from supplementation or fortified foods may become important to understand relative to the possibility of adverse health outcomes. Until the needed research is available, well-informed public policy will be limited by the insufficiency of the evidence for benefit or harm of routine screening and supplementation. The historical focus on concerns for iron inadequacy remains critical for these vulnerable population groups. But, the concerns should now also begin to encompass the parallel interests in ensuring that those who are iron-replete are not put at risk in the broad-brush efforts to avoid iron deficiency. For many understandable reasons, pregnancy and infancy have been almost universally characterized as “automatically” resulting in ID. The situation, especially in more developed regions of the world, many be more nuanced. Research agendas, and in turn public policy, now need to more fully embrace the competing nature of the concerns and find the best balance. Examples of policy decisions that could be better informed by addressing these knowledge gaps and research needs include, but are not limited to clinical guidelines for screening and supplementation; decisions on supplements and food fortification (formulas and infant cereals) such as the type and amount of iron, dietary guidance both for recommended nutrient and food intakes for these two vulnerable populations. The dilemma at present for policy-decisions in developed countries is the duality both of the U-shaped risk and of the nature of iron status of their pregnant women and young children, most of whom are likely iron, but some of whom have sufficient low iron status to be a concern. 

In summary, our knowledge is limited by critical gaps and methodologic challenges that increase the uncertainty in the assessment of iron status across its full continuum in pregnant women and young children whose iron needs are high and in whom adaptations of iron homeostasis may affect their susceptibility to iron excess. Adding to this uncertainty is the lack of cutpoints across the full continuum of iron status that have been related to health outcomes, especially those clinically-relevant and beyond hematologic outcomes. Indicators are also needed that can be appropriated adjusted for or are not affected by inflammation. Advancing our knowledge on the benefits and adverse outcomes of iron supplementation in pregnant women and young children will also inform strong evidence-based policy that ensure sufficiency without excess iron availability in largely iron-replete pregnant women and children in developed countries. 

## Figures and Tables

**Table 1:** Dietary iron reference intake values (mg/day) for pregnant women, infants and young children (12–23 months) in the United States, Canada, Europe, Australia, New Zealand and World.

	Women of Reproductive Age	Pregnant Women	Infants			Young Children
			0 to 6 Months		6 to 12 Months	12 to 23 Months
			0 to 3	4 to 6		
**United States & Canada (IOM ^1^)**	8.1/18 ^2^	22/27 ^2^	0.26 ^3^		6.9/11 ^2^	3/7 ^2^
**Europe**						
EFSA ^4^	7/16 ^2^	7/16 ^2^	Not specified		8/11 ^2^	5/7 ^2^
UK (SACN ^5^)	11.4/14.8 ^2^	11.4/14.8 ^2^	1.3/1.7 ^2^	2.3/3.3 ^2^	6/7.9 ^2^	5.3/6.9 ^2^
**Australia & New Zealand ^6^**	8/18 ^2^	22/27 ^2^	0.2 ^3^		7/11 ^2^	4/9 ^2^
**WHO/FAO ^7^**	19.5/24.5/29.4/58.8 ^8^	Not specified	Not specified		6.2/7.7/9.3/18.6 ^8^	3.9/4.8/5.8/11.6 ^8^

^1^ Institute of Medicine (IOM) [17]; ^2^ Estimated Average Requirement/Recommended Dietary Allowance or Recommended Dietary Intake or Population Reference Intake or Recommended Nutrient Intake; ^3^ Adequate Intake; ^4^ European Food Safety Authority (EFSA) [18]; ^5^ Standing Advisory Committee on Nutrition (SACN) [19]; ^6^ Nutrient Reference Intakes for Australia and New Zealand [20]; ^7^ Food and Agricultural Organization (FAO)/World Health Organization (WHO) [21]; ^8^ Reference Nutrient Intake for 15/12/10/5% bioavailability of dietary iron.

**Table 2:** Recommendations for Iron Screening, Supplementation and Complementary Feeding of Pregnant Women and Infants in the United States, Canada, Europe, Australia, New Zealand and World.

Source	Recommendations	
	Supplement	Supplement and Iron-Rich Complementary Feeding
	Pregnant Women	Infants (0–12 Months)
**United States**		
UpToDate ^1^	15–30 mg/day increase	Supplement 1 mg/kg/day (max. 15 mg/day) breastfed ≥4 months. until consuming sufficient quantities of iron-rich complementary foods
American College of Gynecology ^2^	If iron deficiency anemia (IDA) identified	--
Centers for Disease Control ^3^	Universal (30 mg/day)	Suggest supplement (1 mg/kg/day) breast-fed infants ≥6 months. consuming insufficient iron from supplementary foods (<1 mg/kg/day)
American Academy of Pediatrics ^4^	-	Screen for ID/IDA at 12 months. Supplement (1 mg/kg/day) infants ≥4 months. exclusively breast-fed or consuming >1/2 intake from breast milk until receiving appropriate iron-containing complementary foods
**Canada**		
Infant Feeding Working Group for Health Canada, Canadian Paeditric Society, Dietitians of Canada & Breastfeeding Committee for Canada ^5^		Recommend meat, meat-alternatives & iron-fortified cereals for firs complementary foods at 6 months.
**Europe**		
European Food Safety Authority ^6^	If at risk	-
European Society Pediatric Gastroenterology, Hepatoloy & Nutrition ^7^	No evidence iron supplementation of European women improves iron status of their infants.	No convincing evidence for iron supplements of exclusively breast-fed term infant <6 months. except on individual basis in high risk groups. Recommend iron rich complementary foods (meat, iron-fortified follow-on formulas & iron-fortified foods)
**UK – British**		
Committee for Standards in Haematology ^8^	Supplement if serum ferritin (SF) <30 µg/L	-
**Australia**		
Department of Health ^9^	Do not routinely supplement	-
National Health and Medical Research Council ^10^	-	Introduce first iron-containing nutritious foods (iron-fortified cereals, pureed meat and poultry dishes; care with plant sources such as cooked plain tofu and legumes/beans)
**New Zealand**		
National Women’s Health ^11^	Screen SF & Hb mid 26–28 weeks; supplement low dose (65 mg) if iron deficient and high dose (130 mg) if IDA	-
**World**		
WHO ^12,13^	Supplement (30–60 mg/day)	Iron supplementation (10–12.5 mg/day) in young children (6–23 months) for 3 consecutive months/year. in settings ≥40% anemia prevalence

^1^ UpToDate [29]; ^2^ American College Gynecology [30]; ^3^ Centers for Disease Control [31]; ^4^ American Academy of Pediatrics [25]; ^5^ Infant Feeding Working Group [32]; ^6^ European Food Safety Authority [18]; ^7^ European Society Pediatric Gastroenterology, Hepatology & Nutrition [33]; ^8^ British Committee for Standards on Haematology [34]; ^9^ Australian Department of Health [35]; ^10^ Australian Government National Health and Medical Research Council [36]; ^11^ National Women’s Health [37]; ^12^ World Health Organization (WHO) [6]; ^13^ WHO [5].

**Table 3:** Commonly used indicators of iron status in pregnancy and infancy ^1^.

Indicator	Assesses	Advantages	Limitations
Hemoglobin (Hb)	Anemia	Is commonly available Has low complexity of analytic procedures	Has low specificity and sensitivity Affected by hemodilution in pregnancy and postnatal red blood cell turnover in early infancy May be complicated by certain factors (elevation? age? ethnicity?) Affected by inflammation and obesity
Ferritin (primarily serum, SF)	Size of iron stores	Is commonly available Has World Health Organization (WHO) International Standard Material	Confounded by inflammation
Soluble transferrin receptor (sTfR)	Inadequate tissue availability Iron deficient erythropoiesis	Less affected by inflammation	Has limited availability Exhibits assay differences Lacks a standard reference material (although one is in development)
Ratio of sTfR-to-ferritin (derived using various calculations)	Total body iron stores	Reflects full range of status	Requires two measurements Less affected by inflammation
Transferrin saturation	Iron deficient erythropoiesis	Is commonly available	Varies diurnally and prandially
Erythrocyte protoporphyrin	Iron deficient erythropoiesis		Is reliability infield instrumentation
Hepcidin	Determinant of iron needs and utilization	Is relatively sensitive	Is experimental and under development May possibly be less affected by inflammation

^1^ Adapted from Taylor and Brannon [10].

## References

[1] Aisen P., Enns C., Wessling-Resnick M. (2001). Chemistry and biology of eukaryotic iron metabolism. Int. J. Biochem. Cell Biol..

[2] Wessling-Resnick M. (2017). Excess iron: Considerations related to development and early growth. Am. J. Clin. Nutr..

[3] World Health Organization (2005). World-Wide Prevalence of Anaemia 1993–2005: Who Global Database on Anaemia.

[4] Petry N., Olofin I., Hurrell R.F., Boy E., Wirth J.P., Moursi M., Donahue A.M., Rohner F. (2016). The proportion of anemia associated with iron deficiency in low, medium, and high human development index countries: A systematic analysis of national surveys. Nutrients.

[5] World Health Organization Daily Iron Supplementation in Children 6–23 Months of Age. http://www.who.int/elena/titles/guidance_summaries/iron_children/en/.

[6] World Health Organization Who Recommendations on Antenatal Care for a Positive Pregnancy Experience. http://www.who.int/reproductivehealth/publications/maternal_perinatal_health/anc-positive-pregnancy-experience/en/.

[7] Gupta P.M., Hamner H.C., Suchdev P.S., Flores-Ayala R., Mei Z. (2017). Iron deficiency and adequacy in young children, non-pregnant, and pregnant women in the United States. Am. J. Clin. Nutr..

[8] Milman N., Taylor C., Merkel J., Brannon P. (2017). Iron status in pregnant women and women of reproductive age in Europe. Am. J. Clin. Nutr..

[9] Van der Merwe L.F., Eussen S.R. (2017). Iron status of young children in Europe. Am. J. Clin. Nutr..

[10] Taylor C.L., Brannon P.M. (2017). Introduction to workshop on iron screening and supplementation in iron-replete pregnant women and young children. Am. J. Clin. Nutr..

[11] Fisher A.L., Nemeth E. (2017). Iron homeostasis during pregnancy. Am. J. Clin. Nutr..

[12] Lönnerdal B. (2017). Development of iron homeostasis in infants and young children. Am. J. Clin. Nutr..

[13] Bothwell T.H. (2000). Iron requirements in pregnancy and strategies to meet them. Am. J. Clin. Nutr..

[14] Zhang C., Rawal S. (2017). Dietary iron intake, iron status and gestational diabetes. Am. J. Clin. Nutr..

[15] Paganini D., Zimmermann M.B. (2017). The effects of iron fortification and supplementation on the gut microbiome and diarrhea in infants and children: A review. Am. J. Clin. Nutr..

[16] Vricella L.K. (2017). Emerging understanding and measurement of plasma volume expansion in pregnancy. Am. J. Clin. Nutr..

[17] Institute of Medicine (US) Panel on Micronutrients (2001). Dietary Reference Intakes for Vitamin A, Vitamin k, Arsenic, Boron, Chromium, Copper, Iodine, Iron, Manganese, Molybdenum, Nickel, Silicon, Vanadium, and Zinc.

[18] EFSA Panel on Dietetic Products Nutrition and Allergies (NDA) (2015). Scientific opinion on dietary reference values for iron. EFSA J..

[19] Scientific Advisory Committee on Nutrition (SACN) (2010). Iron and Health.

[20] Australian Ministry of Health Nutrient Reference Intakes for Australia and New Zealand: Iron. https://www.nrv.gov.au/nutrients/iron.

[21] FAO/WHO (2002). Human Vitamin and Mineral Requirements.

[22] Rios E., Lipschitz D.A., Cook J.D., Smith N.J. (1975). Relationship of maternal and infant iron stores as assessed by determination of plasma ferritin. Pediatrics.

[23] Proytcheva M.A. (2009). Issues in neonatal cellular analysis. Am. J. Clin. Pathol..

[24] Dewey K.G. (2013). The challenge of meeting nutrient needs of infants and young children during the period of complementary feeding: An evolutionary perspective. J. Nutr..

[25] Baker R.D., Greer F.R. (2010). Diagnosis and prevention of iron deficiency and iron-deficiency anemia in infants and young children (0–3 years of age). Pediatrics.

[26] Finn K., Callen C., Bhatia J., Reidy K., Bechard L.J., Carvalho R. (2017). Importance of dietary sources of iron in infants and toddlers: Lessons from the fits study. Nutrients.

[27] Siu A.L., U.S. Preventive Services Task Force (2015). Screening for iron deficiency anemia and iron supplementation in pregnant women to improve maternal health and birth outcomes: U.S. Preventive services task force recommendation statement. Ann. Intern. Med..

[28] Siu A.L., U.S. Preventive Services Task Force (2015). Screening for iron deficiency anemia in young children: Uspstf recommendation statement. Pediatrics.

[29] Garner C.D., Post T.W. (2017). Nutrition in pregnancy. Uptodate.

[30] American College of Obstetrics and Gynecology (2008). Acog practice bulletin No. 95: Anemia in pregnancy. Obstet. Gynecol..

[31] Yip R., Parvanta I., Cogswell M.E., McDonnell S.M., Bowman B.A., Grummer-Strawn L.M., Trowbridge F. (1998). Recommendations to prevent and control iron deficiency in the United States. Morb. Mortal. Wkly. Rep..

[32] Infant Feeding Working Group Nutrition for Healthy Term Infants: Recommendations from Birth to Six Months. http://www.hc-sc.gc.ca/fn-an/nutrition/infant-nourisson/recom/index-eng.php.

[33] Domellöf M., Braegger C., Campoy C., Colomb V., Decsi T., Fewtrell M., Hojsak I., Mihatsch W., Molgaard C., Shamir R. (2014). Iron requirements of infants and toddlers. J. Pediatr. Gastroenterol. Nutr..

[34] Pavord S., Myers B., Robinson S., Allard S., Strong J., Oppenheimer C., British Committee for Standards in Haematology (2012). UK guidelines on the management of iron deficiency in pregnancy. Br. J. Haematol..

[35] Australian Department of Health Nutritional Supplements, 10.4.4 Iron Supplementation. http://www.health.gov.au/internet/publications/publishing.nsf/Content/clinical-practice-guidelines-ac-mod1~part-b~lifestyle-considerations~nutritional-supplements.

[36] Australian Government National Health and Medical Research Council Infant Feeding Guidelines: Summary. https://www.eatforhealth.gov.au/sites/default/files/files/the_guidelines/n56b_infant_feeding_summary_130808.pdf.

[37] Auckland District Health Board—National Women’s Health Iron in Pregnancy. http://nationalwomenshealth.adhb.govt.nz/Portals/0/Documents/Policies/Iron%20in%20Pregnancy_.pdf.

[38] Anderson G.W., Frazer D.M. (2017). Current understanding of iron homeostasis. Am. J. Clin. Nutr..

[39] Ross A.C. (2017). Impact of chronic and acute inflammation on extra- and intracellular iron homeostasis. Am. J. Clin. Nutr..

[40] Gordeuk V.R., Brannon P.M. (2017). Ethnic and genetic factors of iron status in women of reproductive age. Am. J. Clin. Nutr..

[41] Rehu M., Punnonen K., Ostland V., Heinonen S., Westerman M., Pulkki K., Sankilampi U. (2010). Maternal serum hepcidin is low at term and independent of cord blood iron status. Eur. J. Haematol..

[42] Domellöf M., Lönnerdal B., Abrams S.A., Hernell O. (2002). Iron absorption in breast-fed infants: Effects of age, iron status, iron supplements, and complementary foods. Am. J. Clin. Nutr..

[43] Leong W.I., Bowlus C.L., Tallkvist J., Lönnerdal B. (2003). Iron supplementation during infancy—Effects on expression of iron transporters, iron absorption, and iron utilization in rat pups. Am. J. Clin. Nutr..

[44] Leong W.I., Bowlus C.L., Tallkvist J., Lönnerdal B. (2003). DMT1 and FPN1 expression during infancy: Developmental regulation of iron absorption. Am. J. Physiol. Gastrointest. Liver Physiol..

[45] Georgieff M.K. (2017). Iron assessment to protect the developing brain. Am. J. Clin. Nutr..

[46] Hoofnagle A.N. (2017). Bioindicator harmonization in clinical research: Making the hard work matter. Am. J. Clin. Nutr..

[47] Pfeiffer C.M., Looker A.C. (2017). Laboratory methodologies for indicators of iron status: Strengths, limitations and analytical challenges. Am. J. Clin. Nutr..

[48] Brannon P.M., Stover P.J., Taylor C.L. (2017). Integrating themes, evidence gaps and research needs identified by workshop on iron screening and supplementation in iron-replete pregnant women and young children. Am. J. Clin. Nutr..

[49] Namaste S.M., Rohner F., Huang J., Bhushan N.L., Flores-Ayala R., Kupka R., Mei Z., Rawat R., Williams A.M., Raiten D.J. (2017). Adjusting ferritin concentrations for inflammation: Biomarkers reflecting inflammation and nutritional determinants of anemia (brinda) project. Am. J. Clin. Nutr..

[50] Rohner F., Namaste S., Larson L., Addo Y., Mei Z., Suchdev P.S., Ashour F., Rawat R., Raiten D.J., Northrop-Clewes C. (2017). Adjusting soluble transferrin receptor concentrations for inflammation: Brinda project. Am. J. Clin. Nutr..

[51] Mei Z., Namaste S.M., Serdula M., Suchdev P.S., Rohner F., Flores-Ayala R., Addo O.Y., Raiten D.J. (2017). Adjusting total body iron for inflammation: Biomarkers reflecting inflammation and nutrition determinants of anemia (BRINDA) project. Am. J. Clin. Nutr..

[52] O’Brien K.O. (2017). Iron status of north american pregnant women: Other evidence from the united states and canada. Am. J. Clin. Nutr..

[53] Daru J., Colman K., Stanworth S.J., De La Salle B., Wood E.M., Pasricha S.R. (2017). Serum ferritin as an indicator of iron status: What do we need to know?. Am. J. Clin. Nutr..

[54] Kemper A.R., Fan T., Grossman D.C., Phipps M.G. (2017). Gaps in evidence regarding iron deficiency anemia in pregnant women and young children: Summary of united states preventive services task force recommendations. Am. J. Clin. Nutr..

[55] Breymann C. (2015). Iron deficiency anemia in pregnancy. Semin. Hematol..

[56] Dewey K.G., Oaks B.M. (2017). U-shaped curve for risk associated with maternal iron status or supplementation. Am. J. Clin. Nutr..

[57] Kozuki N., Lee A.C., Katz J. (2012). Moderate to severe, but not mild, maternal anemia is associated with increased risk of small-for-gestational-age outcomes. J. Nutr..

[58] Lozoff B., Beard J., Connor J., Barbara F., Georgieff M., Schallert T. (2006). Long-lasting neural and behavioral effects of iron deficiency in infancy. Nutr. Rev..

[59] Lozoff B., Goerogieff M. (2006). Iron deficiency and brain development. Semin. Pediatr. Neonatol..

[60] Georgieff M.K. (2011). Long-term brain and behavioral consequences of early iron deficiency. Nutr. Rev..

[61] Cai C., Granger M., Eck P., Friel J. (2017). Effect of daily iron supplementation in healthy exclusively breastfed infants: A systematic review with meta-analysis. Breastfeed. Med..

[62] McDonagh M.S., Blazina I., Dana T., Cantor A., Bougatsos C. (2015). Screening and routine supplementation for iron deficiency anemia: A systematic review. Pediatrics.

[63] Reveiz L., Gyte G.M., Cuervo L.G., Casasbuenas A. (2011). Treatments for iron-deficiency anaemia in pregnancy. Cochrane Database Syst. Rev..

[64] Haider B.A., Olofin I., Wang M., Spiegelman D., Ezzati M., Fawzi W.W., Nutrition Impact Model Study Group (2013). Anaemia, prenatal iron use, and risk of adverse pregnancy outcomes: Systematic review and meta-analysis. BMJ.

[65] McDonagh M., Blazina I., Dana T., Cantor A., Bougatsos C. (2015). Routine Iron Supplementation and Screening for Iron Deficiency Anemia in Children Ages 6 to 24 Months: A Systematic Review to Update the U.S. Preventive Services Task Force Recommendation.

[66] Scholl T.O. (2005). Iron status during pregnancy: Setting the stage for mother and infant. Am. J. Cliln. Nutr..

[67] Pasricha S.R., Hayes E., Kalumba K., Biggs B.A. (2013). Effect of daily iron supplementation on health in children aged 4–23 months: A systematic review and meta-analysis of randomised controlled trials. Lancet Glob. Health.

[68] Lönnerdal B. (2017). Excess iron intake as a factor in growth, infections and development of infants and young children. Am. J. Clin. Nutr..

[69] Singhal A., Morley R., Abbott R., Fairweather-Tait S., Stephenson T., Lucas A. (2000). Clinical safety of iron-fortified formulas. Pediatrics.

[70] Agrawal S., Berggren K.L., Marks E., Fox J.H. (2017). Impact of high iron intake on cognition and neurodegeneration in humans and in animal models: A systematic review. Nutr. Rev..

